# Cylindrical Al Nano-Dimer Induced Polarization in Deep UV Region

**DOI:** 10.1186/s11671-022-03702-7

**Published:** 2022-07-05

**Authors:** Conghui Xu, Jianfeng Wu, Binghuan Chen, Wenyu Kang, Jun Yin, Jing Li

**Affiliations:** Department of Physics, Collaborative Innovation Center for Optoelectronic Semiconductors and Efficient Devices, Jiujiang Research Institute, Pen-Tung Sah Institute of Micro-Nano Science and Technology, College of Chemistry and Chemical Engineering, Xiamen University, Xiamen, 361005 Fujian China

**Keywords:** Surface plasmon resonance, Metal nano-dimer, Polarization, Quadrupole mode

## Abstract

The polarization properties of asymmetric plasmonic nanostructures originating from optical anisotropy show great application prospects in many fields, such as display, sensing, filtering, and detection. Here, we report the realization of polarization control in the deep ultraviolet (UV) region using Al nano-dimer structures. The simulation results indicated that the polarization effect was generated by the modulation of inter-coupling between the quadrupole plasmon resonances of the asymmetric dimer. By further optimizing the size and gap of the dimer, the extinction in the 200-nm deep UV region obtained a polarization ratio of 18%. This research is helpful for understanding the resonance hybridization of high-order surface plasmons in UV region and is of great significance to the emerging polarized micro-nano photonics fields, such as spin optoelectronics and deep UV optoelectronic devices.

**Graphical Abstract:**

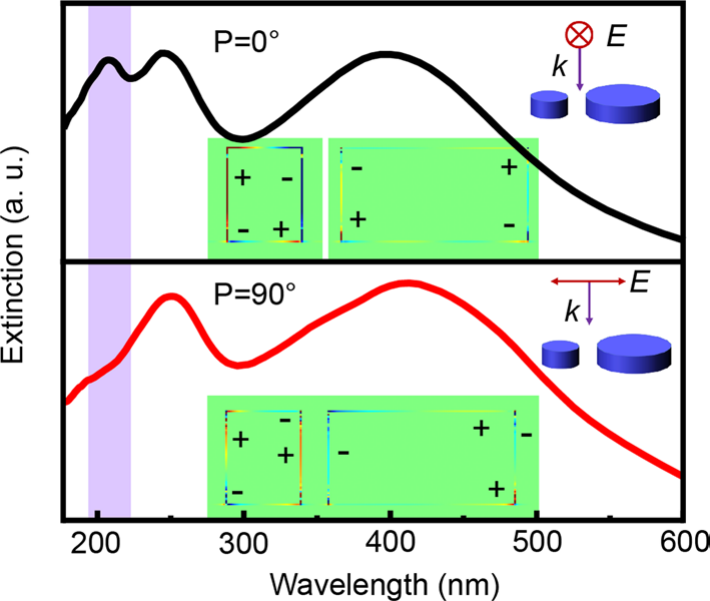

**Supplementary Information:**

The online version contains supplementary material available at 10.1186/s11671-022-03702-7.

## Introduction

Surface plasmons play an important role in many fields owing to their excellent optical properties, including excellent waveguide mode, local-field enhancement, and far-field scattering properties [1, 2]. For example, the waveguide mode surface plamons can be applied to the subwavelength lasers to overcome the diffraction limits of light and improve the radiation efficiency [3]. And localized surface plasmons (LSP) are the more widely used plasmonic mode in Raman scattering [4], photodetection [5], biological imaging [6], solar cells [7], and other optoelectronic fields [8] owing to their simple excitation conditions and superior manipulation performance in local fields. Up to now, the monomer structure of surface plasmons is the mostly studied plasmonic carriers due to their variety of simple preparation methods and the facile modulation of optical properties, such as near-field enhancement and far-field scattering enhancement [9–11]. Other complex nanostructures, such as metal dimers, have also attracted great attention in recent years due to their specific inter-coupling properties [12–15], demonstrating the important promise in areas including shifting of resonance peaks, generating of “hot spots,” and using the quantum effects in the case of extremely small spacing between closely located particles [7, 16].

In addition to these conventional applications of surface plasmons, the structural anisotropy induced optical anisotropy properties also exhibit great advantages in novel light manipulation, such as polarization and chiral optical modulation [17]. However, for the monomer particle, the polarization properties obtained by means of particle anisotropy requires with large size differences between the long and short axes. And this would inevitably lead to an increase in the size of the particles and consequently to a red-shift of the resonance peak away from the deep UV band. For complex structures, current studies also have mainly focused on the visible to infrared band because of the usually adopted inter-coupling effect based on normal dipole mode resonances [18–20]. Research of polarization manipulation in the deep UV region is still devoid. The main challenge is the realizing of efficient dipole resonance and inter-coupling for polarization control in the deep UV region. As a result, the plasmonic based optical anisotropy is still difficult to be used in the applications of wide-bandgap semiconductor based light-emitting devices or single-photon sources [16, 17].

In this work, we proposed the use of an Al nano-dimer structure to excite the polarization-dependent inter-coupling of high-order LSP between the two separated particles in the deep UV region. Theoretical simulations indicated that the coupling effect between the quadrupole resonances dominated this optical anisotropy in the short-wavelength region. And the resonance modes were identified by the charge distributions of the dimer under light excitation. By further optimizing the size and gap of the dimer, an obvious polarization ratio in the extinction spectra has been succesfully obtained at the 200-nm deep UV region. This work provides novel practical guidance for the design of surface plasmon structures with manipulated polarization characteristics at deep UV wavelengths.

## Results and Discussion

A cylindrical Al nanoparticle (NP) dimer was firstly used to investigate the optical anisotropy in the deep UV region, considering that the cylindrical shape is convenient for actual production and is highly symmetrical for resonance mode analysis. The finite-difference time-domain (FDTD) method (FDTD Solutions software from Lumerical, Canada) was performed for the simulation. A perfectly matched layer (PML) was adopted as the boundary condition, the surrounding dielectric environment was set to vacuum (refractive index of 1), and the refractive index of the material referred to the results in the Palik book [21].

**Fig. 1:**
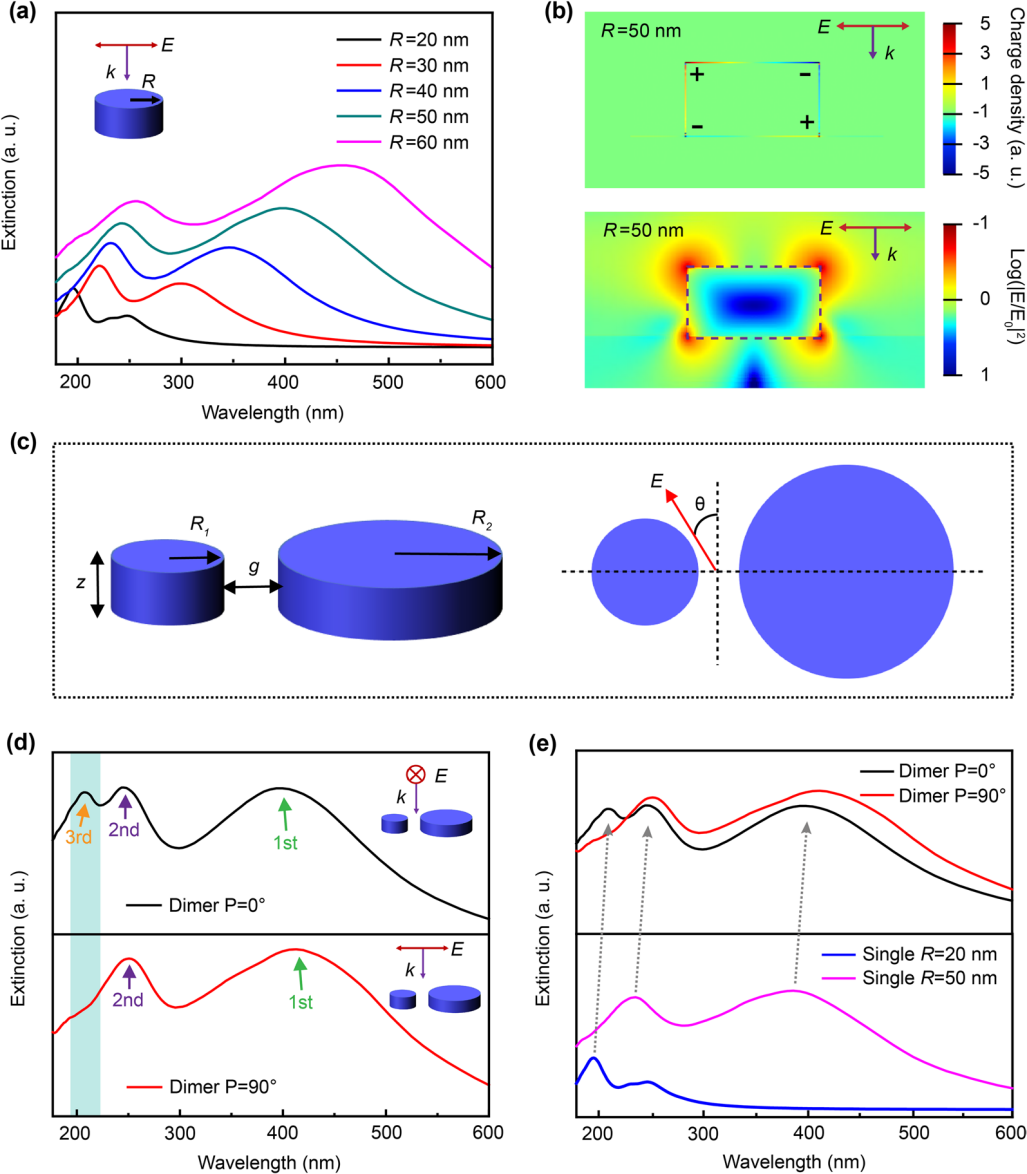
**a** Extinction spectra of Al cylindrical particles with different radius *R* (20, 30, 40, 50, and 60 nm with a height of 50 nm); **b** The extracted charge and near-field distributions for the Al cylindrical particle with *R* = 50 nm when excited at the resonance peak at 234 nm. The distribution patterns indicate the quadrupole mode plasmonic resonance in the deep UV region; **c** Schematic diagram of the dimer structure and the related definition of the parameters; **d** The corresponding extinction spectra of the dimer structure under P = 0° and P = 90° polarized light irradiation, and with comparison to that of **e** the single particles

For a single cylindrical Al NP, two distinct resonance peaks can be well resolved as shown in Fig. 1a, and the resonance in the short-wavelength region can be identified as the quadrupole mode according to the extracted charge and near-field distributions for the Al NP with typical size of radius (*R*) = 50 nm (Fig. 1b) [22, 23]. When the particle size is in the range of 20–60 nm, high-order resonance can well cover the deep UV region and exhibits a typical red-shift phenomenon as the size increases. Meanwhile, it can be found that the quadrupole mode resonances of the cylindrical particles with smaller radius show a narrower full width at half maximum (FWHM) in the deep UV region. Therefore, we selected the appropriate size of the Al NP to adjust the quadrupole resonance in the deep UV region for polarization control via the corresponding structural design. The typical plasmonic cylindrical dimer, as shown in Fig. 1c, where *R*_1_ = 20 nm, *z* = 50 nm, *R*_2_ = 50 nm, and *z* = 50 nm, was used to investigate the optical anisotropic properties. The line across the centers of the two particles was set as the axis, and the angle between the electric field component *E* of the polarized light (P) was defined as *θ*, as shown in Fig. 1c.

The simulated extinction spectra of the dimer structure are shown in Fig. 1d under polarized light irradiation at P = 0° or P = 90° simutaneously compared with that of individual particles (Fig. 1e). It can be seen that the dimer structure had three resonance peaks (located at 208, 250, and 400 nm) when P = 0°, and two resonance peaks (located at 253 and 417 nm) when P = 90°. The three resonant peaks from long to short wavelengths were defined in this work, the first, second, and third resonant peak, respectively. The results indicated that the dimer structure demonstrated an obvious polarization-switchable property in response to incident light when in the short-wavelength band. As shown in Fig. 1d for the third resonant peak, a resonance peak emerged at 208 nm when P = 0°, whereas the peak disappeared at P = 90°, accompanied by the normalized extinction intensity reducing by 18%. Additionally, each resonant peak has a slight redshift compared with that of the single particle, as shown in Fig. 1e. Understandably, the inter-coupling effect between the two Al cylindrical particles produced this red-shift due to the retarded collective oscillations of charges for the whole system when excited by the polarized light parallel to the long axis of the dimer [24].

**Fig. 2:**
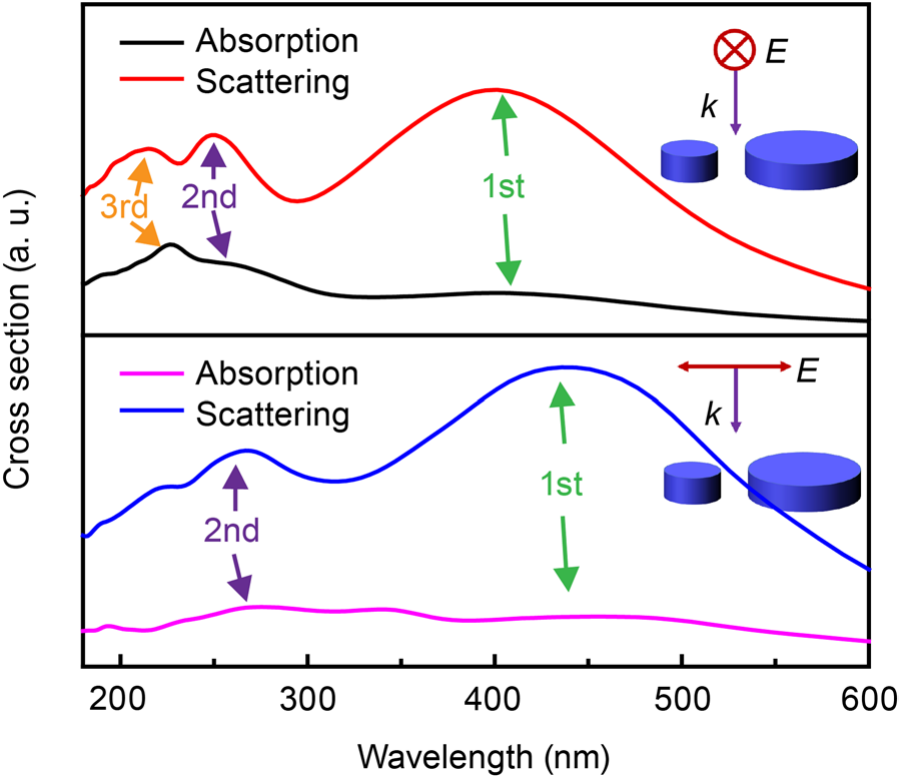
Absorption and scattering spectra of the dimer structure under P = 0° and P = 90° polarized light irradiation

To gain insight into the origin of this resonant property, we extracted the absorption and scattering spectra of the abovementioned dimer structure under P = 0° and P = 90° polarized light irradiation, as shown in Fig. 2. The scattering intensity of the dimer structure was much higher than the absorption intensity under the irradiation of both polarized lights, which was due to the relatively large size of the dimer structure. At the same time, the extinction, scattering, and absorption spectra all shown consistent polarization-switchable properties. This observation might originate from the change in the resonance modes of the dimer structure under different polarized light excitations. In addition, the inter-coupling of the particle resonances leads to a normally observed red-shift of the resonance [13].

**Fig. 3:**
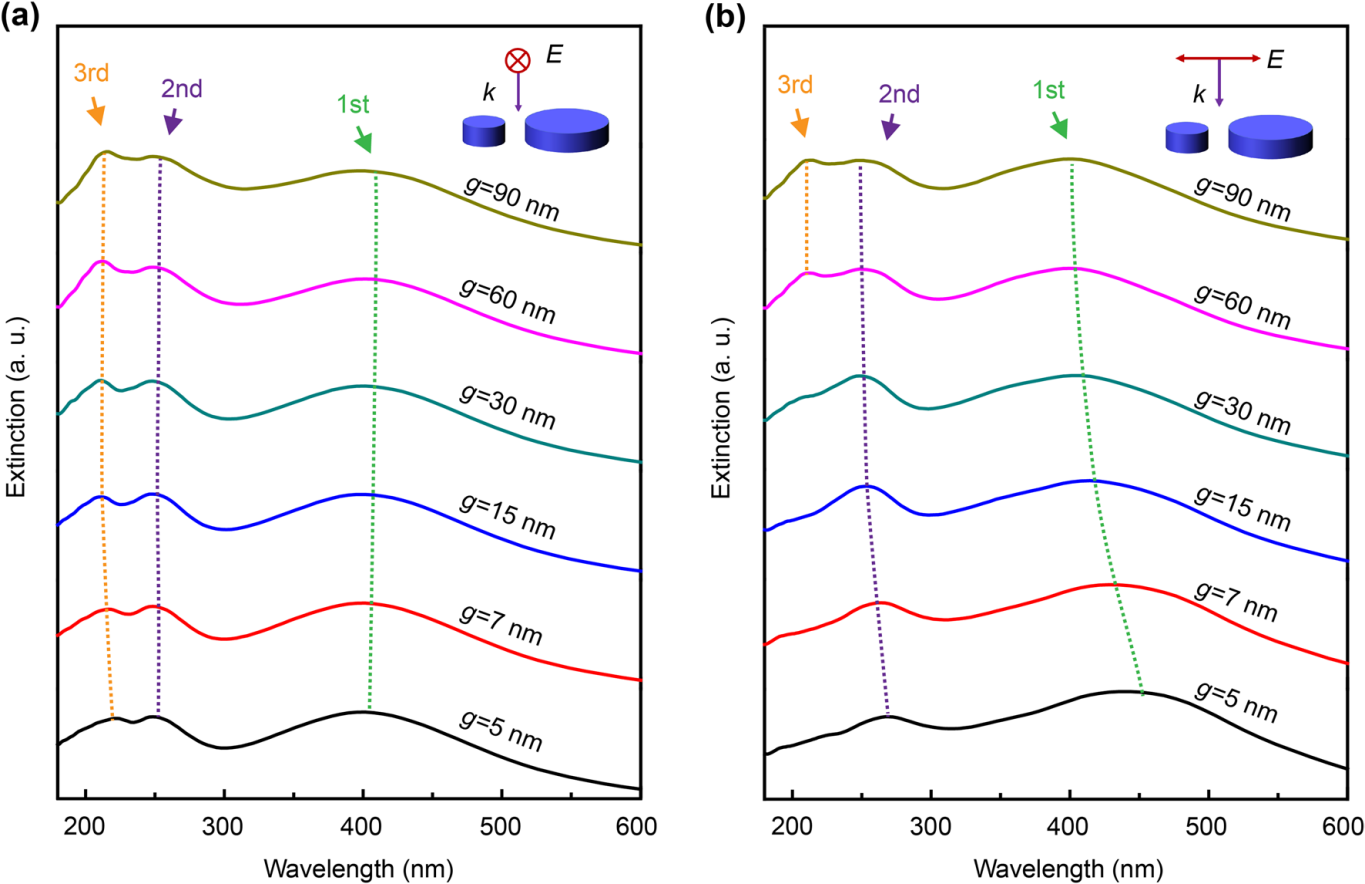
The extinction spectra of the dimer structure with different gaps (*g*) when under **a** P = 0° and **b** P = 90° polarized light irradiation

To verify the dominance of the inter-coupling effect on the polarization property, the plasmonic resonances of the dimer structures with different spacing gaps (*g*) were investigated, as shown in Fig. 3. When P = 0° and *g* increases, the first-order resonance peak (the typical dipole mode resonance near 400 nm), gradually blue shift with correspondingly decreased intensity. While for P = 90°, this amplitude of intensity decreases, and the blue-shift is much larger than that at P = 0°. Here, owing to the introduced electric fields across the two particles in the dimer were independent of each other when P = 0°, the overlapping part of the electric field should be much smaller than that when P = 90°. Thus, the interaction of coupling between the individual particles is weak for the case of P = 0°, and the change in the first-order resonance peak with *g* increasing is not obvious.

However, for the second and third resonance peaks, their interactions were different from those of the dipole resonance. Under the two polarized light irradiations, the second and third resonant peaks shown the same changing trend: as *g* increased, the resonant peak intensity increased. The difference is that the change in the third resonance peak was more evident for the case of P = 90° when compared with that of P = 0° as *g* increased. The resonance peak goes from almost indiscernible to rapidly intensifying as *g* increased when P = 90°, whereas the resonance peak always existed when P = 0°. This property indicates that the resonant coupling of this mode was heavily dependent on the polarization states. That is, the polarization-switchable phenomenon in the deep UV region was realized. As discussed above, a possible reason was that when P = 90°, the overlap between the electric fields was high, and the interaction between the individual particles was stronger. Thus, the changes in the plasmonic resonance properties of the individual particles was obtained.

**Fig. 4:**
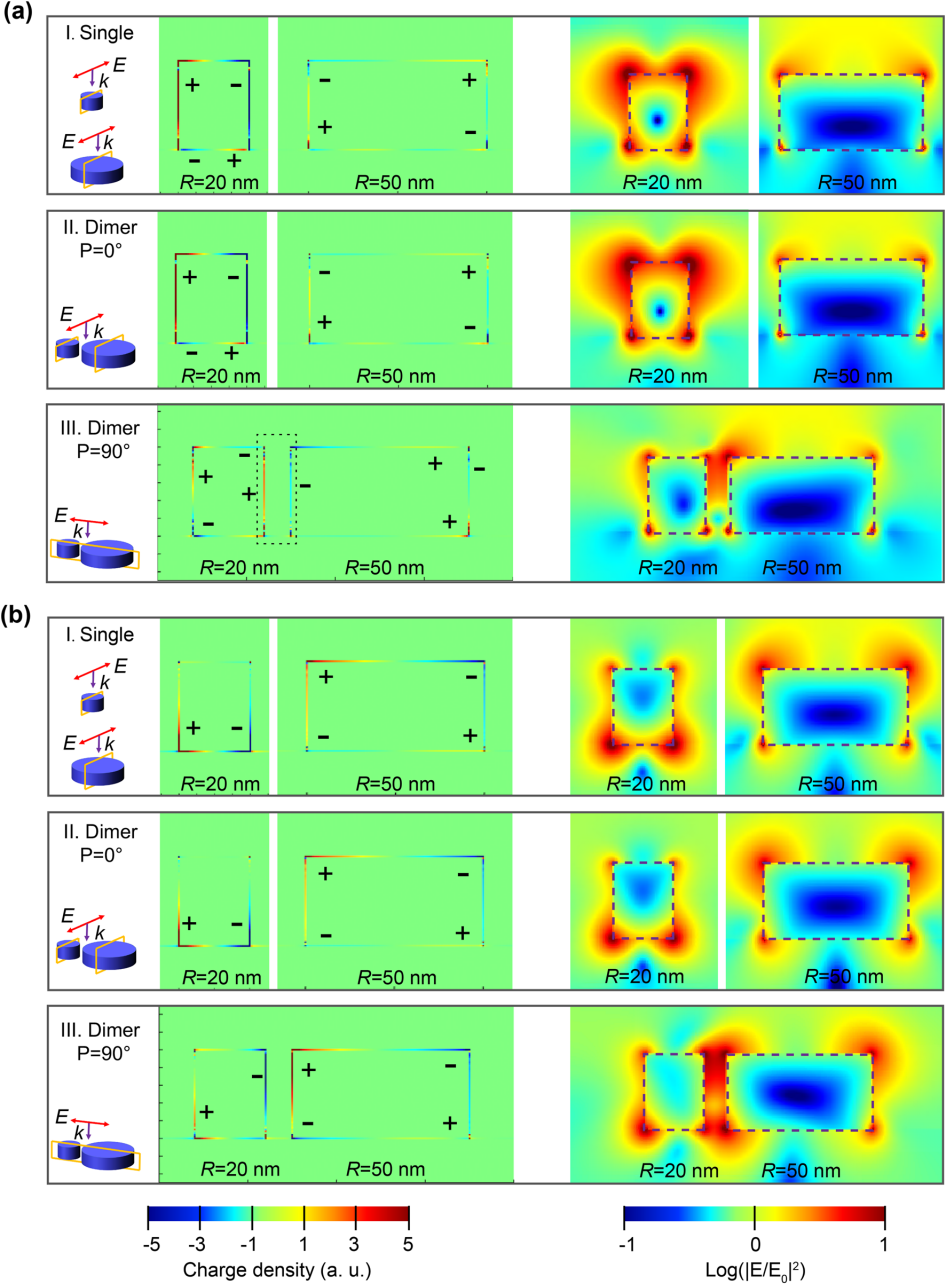
The charge and near-field distributions of the single particle and dimer structure for **a** the third resonance peak (located at 208 nm) and **b** the second resonance peak (located at 250 nm) under different polarized light excitation

To further analyze the generation mechanisms of this polarization-switchable property, the charge distributions of the single particles and dimer structure for the third and second resonance peaks were extracted (*g* = 15 nm), with comparison to their near-field distributions. According to the distribution characteristics of the resonance charges in the cylinder, we selected the charge distribution of the cross-section parallel to the electric field across the center of the cylinder for analysis, as illustrated in Fig. 4. For the third and second resonance peaks, the charge distributions of the dimer structure at P = 0° was similar to that of the individual particles that make up for the dimer (Fig. 4a-I vs a-II and b-I vs b-II). In other words, compared with the single particle, the resonance modes of the two particles in the dimer at P = 0° were almost unchanged; therefore, the dimer exhibits resonance peaks similar to the overlay of the two individual particles at P = 0°.

However, when P = 90°, the excitation electric fields of the large and small particles in the dimer overlapped significantly, and the interaction between the two particles became strong. The charges in the small particle were highly affected by that in the large particle leading to the redistribution of charges in the small particle. As for the third resonance peak and shown in Fig. 4a-III, the part of the large particle that was close to the small particle was negatively charged and attracts the positive charges in the small particle. Subsequently, the charge distribution within the small particle was no longer symmetrical with the simultaneously reduced charge density, resulting in that no efficient plasmon resonance could be formed. These redistributed charges for the dimer hybird were also consistent with plasmonic hybridization characteristics when the two nanoparticles approach within nanometer distances. [13]

This change in the charge distribution of the small particle resulted in the modulated plasmonic resonance, which was different from the situation in their individual forms. As resolved in Fig. 4a-I, the charge distribution for a single particle with *R* = 20 nm had a typical quadrupole mode resonance characteristics: the positive and negative charges in the upper part are distributed in the opposite direction to the lower part. And this was induced by the retardation effect owing to the spatial phase difference of the incident light [23, 25–27]. However, when the dimer was excited at P = 90° (Fig. 4a-III), owing to the modulated charge distribution affected by the neighboring large particle, the asymmetrical charge distribution make it impossible to form a strong resonance with the incident light. Therefore, the third resonance peak of the dimer disappeared. Consequently, the polarization-switchable features of the dimer were obtained. Although the charges’ distribution of the larger single nanoparticle (*R* = 50 nm) also satisfies the quadrupole mode features, the charge density was much smaller than that of the small nanoparticle with radius of 20 nm and it has little contribution to this third resonance peak. Actually, the intrinsic quadrupole resonance peak for the large particle (*R* = 50 nm) was located at around 234 nm (Fig. 1e).

While for the second resonance peak, the small particle (*R* = 20 nm) here shows a dipole mode resonance characteristic (Fig. 4bI–III), whereas the large particle (*R* = 50 nm) shows the typical quadrupole mode resonance. A similar phenomenon of the reformed charge distribution of the dimer can also be resolved when under P = 90°, especially for the small particle with broken symmetry, as shown in Fig. 4b-III. The adjacent part from the large particle shows a mirrored opposite charge distribution induced by the Coulomb force [20, 28]. Similar to the third resonance peak, the weakening of the intensity for the second resonance peak with a decrease in the spacing gap (*g*) should also be caused by the change in the charge distribution of the small particle in the dimer. Nevertheless, it should be noted that the quadrupole plasmonic resonance from the large particle dominated this second resonance peak, and the charges’ distribution was almost unchanged under the different polarized states of incident light (Fig. 4b-III). Thus, polarization-sensitive performance has not been observed at this resonance peak.

The extracted near-field distributions also matched well with the corresponding charge distributions discussed above. Especially, for the dimer structure when excited by the incident light polarized at P = 90° (Fig. 4a-III), significantly declined local-field intensity happened on the third resonance peak for the small particle when compared with that at P = 0°, which was consistent with the reduced charge density under the excitation. The inter-coupling effect within the dimer also can be well resolved from the extracted near-field patterns (Fig. 4a-III and Fig. 4b-III), showing as the intensified local-field intensity within the gap of the dimer.

In order to demonstrate the advantages of the introduced asymmetric structure in polarization control when compared with the generally used symmetrical dimers, the evolution of the polarization properties from symmetrical structure to asymmetric structure has been further analyzed, as shown in Additional file 1: Figure S1. The results indicated that when the dimer was in symmetrical configuration (such as *R*_1_ = *R*_2_ = 20 nm), the quadrupole mode resonances of the Al cylindrical particle both can be excited under P = 0° and P = 90°, and the polarization-sensitive property could not be realized in the symmetrical dimer. When the size of the large particle increased to a suitable value (such as *R*_2_ = 50 nm, the typical asymmetric configuration of the dimer), the polarization-sensitive properties were obtained due to the enhanced tailoring of quadrupole plasmonic resonance for the small particle by the neighboring larger one.

**Fig. 5:**
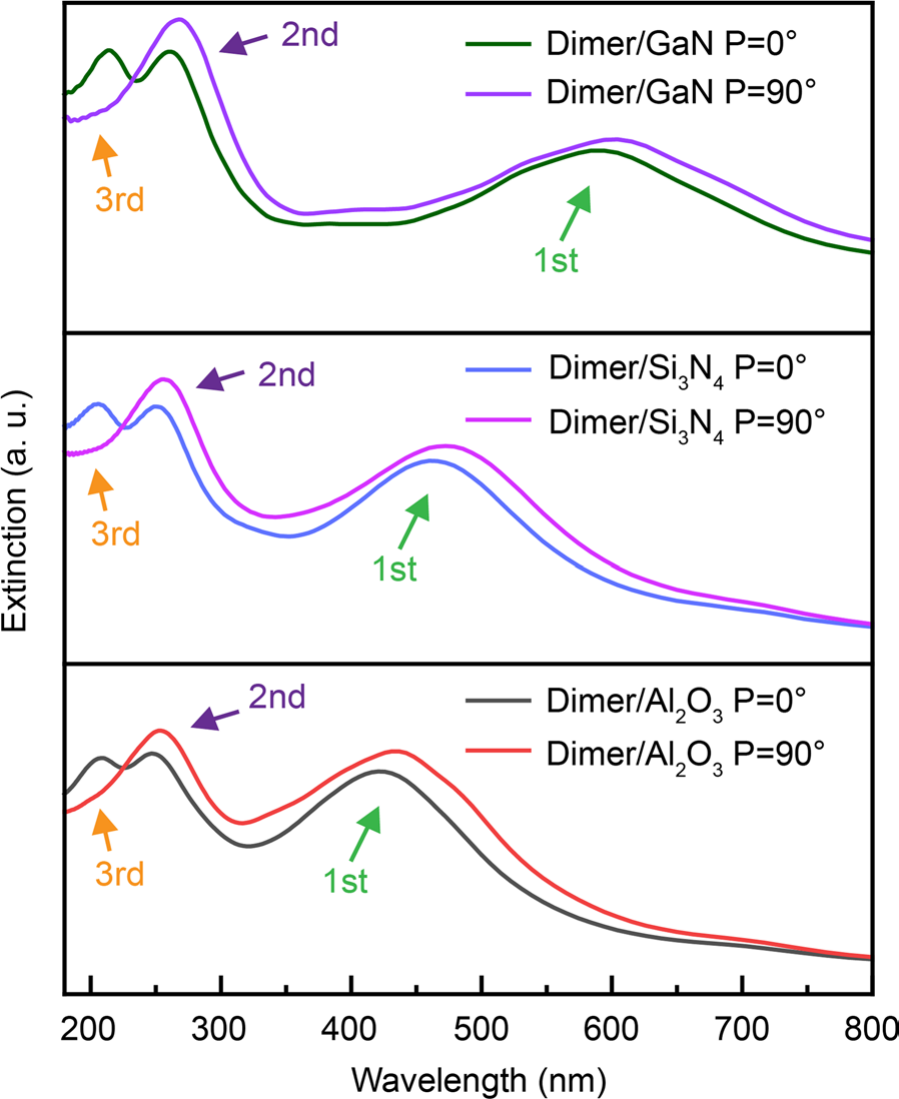
The extinction spectra of the dimer structure under P = 0° and P = 90° polarized light irradiation on Al_2_O_3_, Si_3_N_4_, and GaN substrates, respectively

Considering that the substrate has a large influence on the resonance of the LSP, [11, 29] we also verified this polarization-switchable property on different semiconductor substrates. Three common semiconductors were selected, in order of refractive index from small to large, Al_2_O_3_, Si_3_N_4_, and GaN, respectively. [30, 31] It could be seen that the dimer structures all had a similar phenomenon on the different substrates, as shown in the extinction spectra of Fig. 5. As the refractive index of the substrate increases, the second and third resonant peaks hardly moved, whereas the dipole resonant peak showed a significant red-shift. That is, the quadrupole resonance, which was highly localized near the metal particles, was less affected by the external medium, whereas the dipole resonance, which is mainly outside the metal particles, was greatly affected by the external medium [32, 33]. This also indicates that the polarization property based on the inter-coupling effect of higher-order plasmonic modes was more universal. In addition, further simulation results based on the asymmetric dimer arrays demonstrated that the polarization property can be additionally manipulated by the periodic structure when considering the interdimer coupling (Additional file 1: Figure S2) [15].

## Conclusions

In this work, an asymmetric dimer structure was proposed to realize polarization-switchable properties in the deep-ultraviolet wavelength region by utilizing polarization-sensitive plasmonic coupling modes between the individual particles of the dimer. Theoretical simulations indicated that the redistribution of the collective resonance electrons dominates the polarization-switchable property when was excited by polarized light across the asymmetric dimer. And this property has been intuitively demonstrated by the extracted charge distributions under different resonance modes. By optimizing the coupling distance of the cylindrical Al metal structure, a polarization ratio of up to 18% was successfully obtained in the deep UV region. This research shows great potential for the development of new types of plasmonic-based polarization control methods and their applications in polarization and chiral optoelectronic devices.

## Supplementary Information

**Additional file 1 Fig. S1**: Extinction spectra evolution for the dimer from symmetrical configuration to the asymmetrical structure.** Fig. S2**: Extinction spectra of the dimer array structure under different polarized incident light.

## Data Availability

Not applicable.

## Abbreviations

UV: Ultraviolet
LSP: Localized surface plasmons
NP: Nanoparticle
FDTD: Finite-difference time-domain
PML: Perfectly matched layer
FWHM: Full width at half maximum
