# Supplementary material for: Cylindrical Al Nano-Dimer Induced Polarization in Deep UV Region
Source: Nanoscale Res Lett. 2022 Jul 5;17:62. doi: 10.1186/s11671-022-03702-7 (PMC9256893; doi:10.1186/s11671-022-03702-7)
Supplement: Supplementary file 1 — Additional file 1 Fig. S1: Extinction spectra evolution for the dimer from symmetrical configuration to the asymmetrical structure. Fig. S2: Extinction spectra of the dimer array structure under different polarized incident light. [file 11671_2022_3702_MOESM1_ESM.docx]

**Additional file 1 for**

Cylindrical Al nano-dimer induced polarization in deep UV region

Conghui Xu,^1^ Jianfeng Wu,^1^ Binghuan Chen,^1^ Wenyu Kang,^1*^ Jun Yin,^1*^ and Jing Li^1^

Collaborative Innovation Center for Optoelectronic Semiconductors and Efficient Devices, Jiujiang Research Institute, Department of Physics, Pen-Tung Sah Institute of Micro-Nano Science and Technology, College of Chemistry and Chemical Engineering, Xiamen University, Xiamen, Fujian 361005, China

Correspondence: wykang@xmu.edu.cn and jyin@xmu.edu.cn


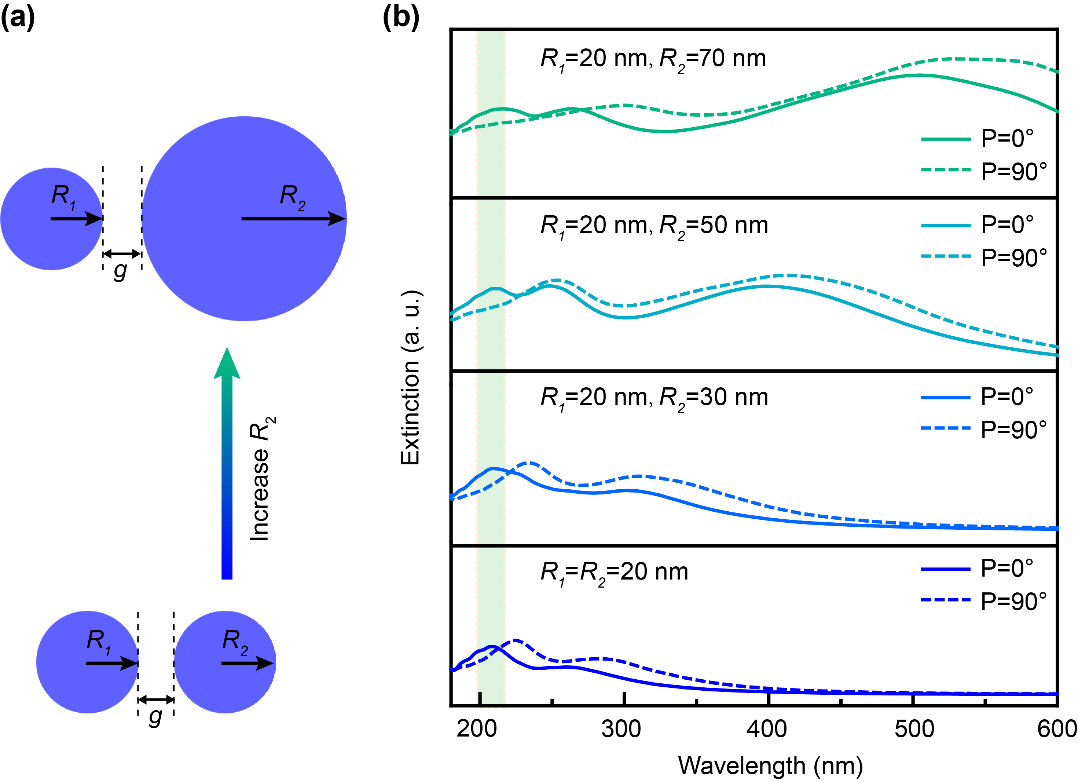


**Figure S1**. (a) Schematic illustration of the dimer structure with increased radius (20 nm to 70 nm) for the cylindrical particle. (b) The simulated extinction spectra of the dimer structure with different *R_2_* under polarized light irradiation at P=0° (solid line) or P=90° (dash line) when the size of small particle and the gap of two particles remain the same (*R_1_*=20 nm and *g*=15 nm).

Here, the size of the neighboring particle was changed from *R*_2_=20 nm (symmetrical structure) to *R*_2_=70 nm (asymmetric structure) for investigation. It can be seen that when the dimer was in symmetrical configuration (*R*_1_=*R*_2_=20 nm), the quadrupole mode resonances of the Al cylindrical particles were well excited both under P=0°and P=90°. And the polarization-sensitive property was not realized in this symmetrical dimer while only a less pronounced redshift was observed when the excitation light changed from P=0° to P=90°. However, when the size of the large particle increased to *R*_2_=50 nm (the typical asymmetric configuration), three resonance peaks were resolved in the extinction spectra at P=90°, and the third resonance disappeared at incident light P=0°. That is, the polarization characteristics have been realized. Additionally, when the size of the large particle continually increased (*R*_2_=70 nm), the redshift for the high-order resonance in the deep-UV region was observed. This should be possible due to the overly altered resonance environment near the large particle.


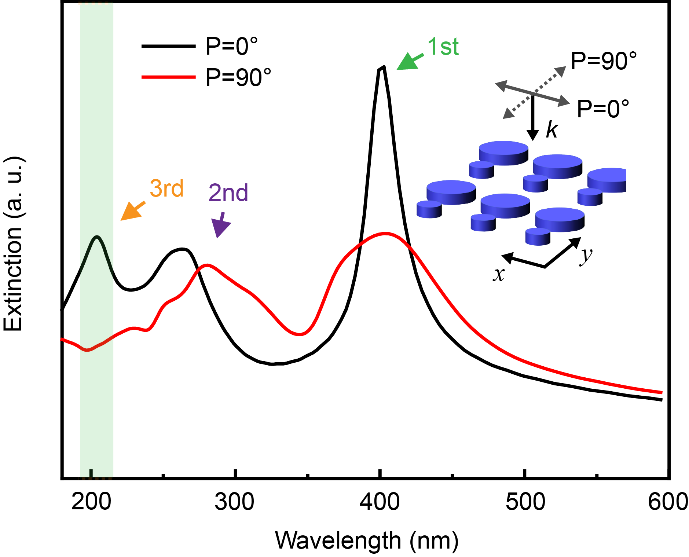


**Figure S2**. Extinction spectra of the dimer array structure under the incident light polarized at P=0° and P=90°. The dimer arrays were schematically illustrated in inset. The configuration of the dimer structure was the same as that investigated in the main text (*R_1_*=20 nm, *R_2_*=50 nm, *z*=50 nm and *g*=15 nm), and the periodicities of the array along *x* and *y* axis were set to be 290 nm and 230 nm, respectively.

As seen from the figure, the extinction spectra for the dimer array structure show the similar polarization properties as that for the single dimer simulated in Figure 1(d-e) of the main text: Three distinct resonance peaks were resolved from the spectra under the excitation of P=0°, and the third high-order resonance disappeared when the polarization of incident light changed from P=0° to P=90°. Compared with the single dimer, it should be noticed that the polarization ratio (~30%) for the plasmonic dimer has been further improved when in the configuration of periodic arrays, which should be originated from the additional manipulation from the inter-dimer coupling effect within the arrays.
